# Motives of Therapists for Using Routine Outcome Monitoring (ROM) and How it is Used by Them in Clinical Practice: Two Qualitative Studies

**DOI:** 10.1007/s10488-024-01374-2

**Published:** 2024-04-08

**Authors:** Shaghayegh Azizian Kia, Lisette Wittkampf, Jacobine van Lankeren, Pauline Janse

**Affiliations:** 1 Pro Persona Research, Wolfheze, The Netherlands; 2 Behavioural Science Institute, Radboud University, Nijmegen, The Netherlands

**Keywords:** Progress Feedback, Routine Outcome Monitoring, Measurement Based Care, Qualitative Analysis, Implementation

## Abstract

Despite its demonstrated value, many mental health institutions struggle to implement progress feedback effectively. There is also insufficient information about how therapists utilize progress feedback. To gain more insight, two qualitative studies were conducted. The first study compared the attitudes and motives of therapists who used and those who did not use progress feedback. The second study examined how psychologists incorporated progress feedback into their practice. In total, 23 therapists were interviewed, and the data were analyzed using thematic analysis. The first study found that almost all the therapists had a positive attitude about progress feedback. Those who did not use it indicated reasons such as a heavy workload and patient-related factors, and they also lacked sufficient information about the potential benefits of progress feedback. The second study revealed four major ways in which the therapists utilized feedback, namely: supporting actions to discuss progress feedback, discussing progress feedback with patients, modifications in the ongoing treatment, and peer consultation. However, discussions during peer consultations about using progress feedback for patients who were not benefitting from treatment and how the treatment might be adjusted accordingly were often lacking. In conclusion, it is crucial that training and education be provided to therapists on how to use progress feedback effectively. Having regular discussions about progress feedback during peer consultations could also facilitate its integration into clinical practice.

**Supplementary Information:**

The online version contains supplementary material available at 10.1007/s10488-024-01374-2.

## Introduction

Approximately 50-to-66% of patients receiving psychological treatments improve (Barkham & Lambert, 2021; Castonguay et al., 2021), yet a considerable proportion of patients do not benefit. Moreover, the percentage of clients who deteriorate during therapy ranges from 5 to 14% (Boswell et al., 2015; Lambert & Shimokawa, 2011). Thus, treatment might even be harmful for some clients. Therapists often fail to accurately identify clients at risk of not benefiting from treatment using only clinical observation (Garb, 2005; Hatfield et al., 2010). Furthermore, when a treatment stagnates, therapists often underestimate patients’ deterioration (Hannan et al., 2005) while overestimating their skills (Walfish et al., 2012). These statistics underscore the value of using objective measurements to improve treatment efficiency. One way in which treatment could be improved would be to identify early in the treatment when a patient is not making sufficient progress (Wampold, 2015). This might be achieved through the use of progress feedback (PF), which is also called Routine Outcome Monitoring (ROM), and Measurement Based Care (MBC). PF refers to regularly measuring patients’ progress using standardized measures (De Jong et al., 2012), evaluating the progress with the patient, and, when necessary, adapting the treatment accordingly (Lambert, 2010). These goals might be achieved by using motivational interviewing to discuss patients’ concerns and thereby strengthen the therapeutic alliance (Mütze et al., 2021). Using PF might correct therapists’ potential blind spots (Macdonald & Mellor-Clark, 2014), and less effective treatments might be adjusted in consultation with the patient and by objectively measured treatment responses (Janse & De Jong, 2020).

Recent meta-analyses showed that the use of PF had positive effects on symptom reduction and the reduction of treatment dropouts among adults, particularly among those who failed to progress, i.e. those who were not on track (NOT) (De Jong et al., 2021; Rognstad et al., 2023). The use of PF can also help to improve therapists’ performance. Delgadillo et al. (2022), for instance, showed that PF narrowed the gap in treatment outcomes between the most and least effective therapists. However, the effects of PF are small overall, and more knowledge is still needed on factors that influence whether or not PF is effective and how its uptake can be improved (McAleavey et al., 2024).

Despite the demonstrated benefits of PF, therapists do not make sufficient use of it or use it inconsistently in their treatment of adults. The use of PF has been shown to vary from 12-to-14% in North America (Ionita & Fitzpatrick, 2014; Jensen-Doss et al., 2018; Lewis et al., 2019) to 69% in Australia (Chung & Buchanan, 2019). It appears, therefore, that many therapists are missing opportunities to optimize their treatments. Accordingly, acquiring more insight would appear to be essential for effectively implementing PF and improving treatment efficiency, duration, and outcomes (Sapyta et al., 2005).

Therapists’ implementation of progress feedback (PF) in their therapy is influenced by both their attitudes (Jensen-Doss et al., 2018) and their motives. Therapists have highlighted the benefits of using PF, such as its positive effects on treatment monitoring, evaluation, reflection, and the early detection of stagnation (Norman et al., 2014; Sharples et al., 2017; Unsworth et al., 2012), and it fosters patients’ dialogue and collaboration with their therapist (Delgadillo et al., 2017; Unsworth et al., 2012). Nevertheless, therapists also cite reasons for not using PF. For example, some therapists fear that using PF may worsen the therapeutic relationship, depersonalize their contacts with patients, and consume session time (Norman et al., 2014; Sharples et al., 2017). Some therapists have felt that the questionnaires used have not aligned well with their patient demographics (Sharples et al., 2017), and concerns were also expressed regarding personal evaluations by the treatment organization (Unsworth et al., 2012). The time required to learn PF systems and the pressure on discussing PF during consultation sessions were also mentioned as disadvantages (Boswell et al., 2015; Delgadillo et al., 2017; Norman et al., 2014; Sharples et al., 2017; Unsworth et al., 2012). Despite the extensive research on therapists’ attitudes about PF and their motives for using it, no study has specifically compared therapists who use PF and those who do not use it to determine differences between the two groups.

In addition, other factors require further research. These include the mechanism by which PF works (Wampold, 2015; Whitcomb et al., 2018) and exactly how therapists can utilize PF effectively. Brooks Holliday and colleagues (2021) found that best practices included providing patients with a strong rationale for using PF, discussing results frequently, and using graphs to visualize results. Låver and colleagues’ (2023) recent review and meta-analysis of qualitative studies found that patient-reported data were used to (a) objectify patients’ progress, (b) enhance self-awareness and initiate reflection, and (c) facilitate patient-therapist interactions. Although there is a small, but growing literature on this topic, more information is needed about how therapists are using PF in their clinical practice and what steps they take to improve the effectiveness and uptake of PF.

In short, there are various questions surrounding the use of PF. Specifically, (a) why, within the same organization, do some therapists use PF but others do not, and (b) among the therapists who use PF, how do they do so? To answer these questions, two qualitative studies were conducted. In Study 1, we identified the characteristics of psychologists who use and those who do not use PF. In Study 2, we examined how psychologists make use of PF in order to adjust their interventions or treatment plans, and whether the information obtained from the use of PF was discussed during peer consultations. Our aim was to determine how the information obtained from these two studies could be utilized to better implement PF in mental health care and lead to improved treatment outcomes and reduced dropout rates.

## Method

### Design, Participants, and Setting

Two qualitative studies were run in which semi-structured interviews were conducted by psychologists working in a large mental-health facility. Different participants were recruited for Study 1 and Study 2, but all participants were trained psychologists who were employed by the same mental health organization in the Netherlands. These therapists worked in teams that provided specialized outpatient care for individuals who were coping with psychological disorders, in particular anxiety and mood disorders. The treatment provided by each team followed multidisciplinary guidelines, and all therapists had a cognitive-behavioral orientation.

Within the mental health organization, questionnaires were available digitally and were usually sent to patients automatically. However, the questionnaires were also available in paper-and-pencil format for patients who preferred them. The frequency of administration varied from weekly to every six weeks and depended on the questionnaire. Another option was for the therapists to send the questionnaires manually to their patients. Scores from the questionnaires were displayed in the electronic patient record (EPR). E-learning was available, whereby the therapists learned how to administer the questionnaires and view the results. The EPRs included descriptions of PF for the most used forms, such as the intake form and the treatment plan, and various letters addressed to patients. Therapists could direct questions they might have to a department dedicated specifically to PF. There was an Intranet page where information about questionnaires that used PF could be shared.

As further background for the two studies, it should be noted that the use of progress feedback is mandatory in clinics that provide psychotherapy in the Netherlands. In recent years, this has generated discussions regarding the burden for patients and therapists and patient privacy. This concern has led to some resistance in the use of PF in the Netherlands (Van Os et al., 2017).

The participants in Study 1 included one male therapist and twelve female therapists. Seven of these therapists were designated as *users* of progress feedback because their patients regularly completed questionnaires throughout their treatment and engaged in discussions related to outcomes. Six participants were designated as *non-users* because they did not incorporate any measurements into their practice. Instead, they used the questionnaires only at the start or the end of the treatment and did not discuss the results with the patients. The participants in Study 2 included seven female and three male therapists. See Table 1 for a summary of the characteristics of the participating therapists.

**Table 1:** Participant characteristics of Study 1 and Study 2

	Study 1		Study 2
	Users *N*	Non-Users *N*	*N*
Gender			
Male	0	1	3
Female	7	5	7
Age Range			
20–30	4	2	3
31–40	2	2	3
41–50	0	0	3
51–60	0	0	0
> 60	1	2	1
Years of Experience			
0–10	4	3	5
11–20	2	2	4
21–30	0	0	0
31–40	1	1	1

### Instruments

For both studies, the guidelines for conducting the interviews were developed based on prior research, feedback from the supervisory team, and input from a focus group. The focus group was conducted to gain further insight into the subject and to generate additional ideas for the interview guidelines. The focus group included both users and non-users of PF. Altogether there were seven female psychologists, including one psychologist trainee, one master-level psychologist, and five registered healthcare psychologists. The participants were asked to discuss the use of PF among themselves. Based on the insights gained from the focus group, relevant topics were integrated into the interview guidelines. The main interview guidelines are shown in Tables 1 and 2 of the supplemental materials. Following the discussion of PF, two pilot interviews were conducted to refine the interview guidelines. Participants in the focus group were excluded from the subsequent interviews.

### Procedure

#### Recruitment

During team meetings, the researchers presented information about the study, and participants were recruited. Subsequently, psychologists in the respective teams were asked to complete (a) a short questionnaire to indicate their willingness to participate in the study and (b) a brief questionnaire to indicate their gender, age, discipline, location, years of work experience, theoretical orientation, and use of progress feedback (PF). There were four options to indicate their prior use of PF:

*yes*, very regularly (I discuss results from the questionnaire with my patient);

*yes*, regularly (I discuss the baseline, intermediate, and final results with my patients);

occasionally (for example, I discuss only the baseline and/or final results) and

look only at the other results for my own benefit; and.

*no*.

In total, 60 psychologists completed the questionnaire. They included 37 (61.7%) psychologists who expressed an interest in participating; nine (15%) psychologists who were uncertain and requested further contact; and 14 (23.3%) psychologists who declined to participate. Based on results from the questionnaires, sample matrixes were constructed for both studies to insure that there would be a diverse mixture of disciplines, locations, ages, experience, and gender. Based on the information in the matrixes, psychologists were approached about participating. Initially, primarily only those who were already using PF indicated their willingness to participate. This, however, inadvertently introduced a selection bias into Study 1. Subsequently, therefore, recruitment was focused on actively recruiting psychologists who were not already using PF. This endeavor resulted in a sufficient response rate from non-users of PF.

Prior to the interviews, the participants were informed about the purpose and procedure of the research. Participants’ written informed consent was obtained before they enrolled. Both studies were reviewed and approved by the Ethics Committee for Social Sciences (ECSW) at Radboud University, Nijmegen (Reference Numbers ECSW-2020–123 and ECSW-2021–055).

Each study continued until the interviews ceased to yield new insight, a phenomenon known as *thematic saturation*. Saturation is typically achieved after five to 15 interviews, and these are followed by two additional interviews to confirm that saturation had been achieved (Baarda et al., 2013).

### Interviews

The research was conducted during the COVID-19 pandemic. Accordingly, all but one of the interviews were conducted via video conferencing (using Zoom software). The researcher documented the context (location, time, conditions) in which the interviews took place and any observations made during the interviews. The interviews were audio-recorded and stored in a secure location at the mental health institution, thus complying with the General Data Protection Regulation (GDPR). The interviews lasted between 35 and 66 min, with an average duration of 43 min. A summary of each interview was transcribed and presented to the therapist, who was asked to read and confirm whether the interviewer’s interpretations accurately reflected his or her opinions and motives (i.e., *member checking* was observed). Almost all the therapists recognized themselves in the summaries and did not make any additions. One therapist, however, did not respond due to a prolonged illness, and one had some practical clarifications, but these did not alter the coding. After each interview, the audio-recordings were transcribed in their entirety. To enhance the readability of the transcripts, filler words and repetitions were omitted. To maintain transparency, all of the steps in the procedure were documented in research memos.

### Research Team

The first two authors conducted and analyzed the interviews. Both of them were registered psychologists and in training to become clinical psychologists working at the mental health institution where the studies were conducted. Both had experience in and were knowledgeable about working with PF. In preparation for the interviews, they together conducted the focus group and recruited the participants. They first coded the interviews independently of each other and subsequently coded each other's interviews. Together they discussed the codes assigned to reach a consensus. Additionally, there was a supervision team that included the third author (a nurse specialist and a qualitative researcher) and the fourth author (a clinical psychologist and senior-level researcher). There was no direct relationship between the researchers and the participants.

### Data Analysis

Thematic analysis was used to analyze the transcripts. This provided flexibility in interpretation of the data and involved six steps as follows: familiarization, coding, generating themes, reviewing and refining, defining and naming themes, and reporting (Hennink et al., 2020). In the first step, each interview was transcribed, read, and re-read for the scorer to become familiar with the data. Subsequently, the data were coded, whereby each phase was assigned a distinct coding method: open, axial, or selective coding (Braun & Clarke, 2006). During the open-coding phase, the researchers partitioned the transcripts into smaller textual units, each of which was relevant for addressing the research question. Labels were then assigned to each text fragment. A second, independent coder then assigned open codes to each interview, after which the two coders compared the codes. In case of discrepancies between the two coders, the text fragment was discussed with the supervisory team to reach a consensus. After the fourth interview, the comparison of codes led to the merging of overlapping codes into axial codes, thereby bridging the gap between open and selective coding. The axial coding process was reiterated after the eighth and 12th interviews. Within the coding process, the implicit meaning of the text was sought, also called latent coding. Further to ensure transparency, an audit trail was made to document each step in the process. After axial coding had been completed, selective coding took place. In this phase, important overarching themes were identified to integrate the data so that the primary research question could be answered. During the analysis, a colleague with expertise in qualitative research and who had not been involved in the studies, was asked to critically review the results obtained from the interviews (i.e., peer debriefing).

Both inductive and deductive methodologies were employed to analyze the data. Informed by the existing literature, themes were derived from the data. This approach was used for all of the interviews. In the fourth step (reviewing and refining), the dataset was revisited, and the themes were compared with the data to ensure that the themes accurately represented the data. The themes were then finalized. Throughout the analysis, meetings were held with the research team to discuss questions related to all the steps in the thematic analysis. The software program AtlasTi (version 23) was used to store and analyze the data, which involved coding and writing memos.

## Results

### Study 1

Both the therapists who used PF (the *users*) and those who did not (*non-users*) expressed a predominantly positive attitude about PF, although one *non-user* was ambivalent. The negative attitude was mainly due to this therapist’s difficulty working with questionnaire scores and the burden to patients of having to frequently complete the questionnaires. Nevertheless, this *non-user* acknowledged the value of using PF to support the evaluations, and the objectification of symptoms and monitoring of treatment progress that PF enabled.

Overall, the therapists named various reasons for using or not using PF, and they identified seven themes: *supportive of treatment*, *perceived disadvantages*, *organizational factors*, *patients’ responses and characteristics*, *therapists’ knowledge about PF*, *therapists’ need for information*, and the *computer system*.

### Theme One: Supportive of Treatment

This theme indicates therapists’ belief that PF had positive effects on the treatment. All the therapists indicated that PF facilitated the treatment, with the *users* more frequently mentioning this than the *non-users*. Participants who used PF frequently mentioned that PF helped them to monitor the treatment and to adjust it accordingly. They emphasized PF’s positive contributions to patients’ collaboration and their motivation. Most of these therapists also stated that PF provided an opportunity to engage in discussions with the patients and to align themselves with the patients. They also often reported that PF served as confirmation that they were on the right track:

"But also for myself, it gives me confidence that I am doing the right thing. I must admit, as a novice psychologist, I sometimes doubt myself and wonder if I am doing it right [laughs]. And when I see such a change in the questionnaire, I think, oh well, maybe it is related to the treatment." [Participant 10, user, female, age range: 20-to-30 years]

Both *users* and *non-users* indicated that PF provided important information about their patients, which had not readily been available previously:

"Especially when I just started working in my team and did not have much experience with autism, it was not always visible for me when people were quite depressed. But then, when you look at the questionnaire, you see that they are severely depressed. I was shocked because I thought, wow, they are so depressed; it is going really badly, and I cannot see it, I cannot judge it." [Participant 4, user, female, age range: 20-to-30 years]

Therapists in both groups mentioned that PF helped keep the treatment focused, objectify symptoms, and support the treatment evaluation. Most of the therapists specifically mentioned the added value of a symptom-oriented questionnaire for PTSD (PTSD Checklist for DSM-5, PCL-5) and a general questionnaire related to complaints, interpersonal relationships, and social roles (Outcome Questionnaire-45).

### Theme Two: Perceived Disadvantages

This topic deals with the perceived disadvantages of PF. Most of the therapists believed that PF was a burden for patients, particularly when they had many standardized questionnaires to complete. They also mentioned that the questionnaires did not always measure what they wanted to know because the treatment focus was not aligned with the purpose of the questionnaire. Adopting PF required time and effort. Those who did not use PF perceived that it had more disadvantages than those who did use it. Almost all the *non-users* had many work pressures, and PF was given lower priority than their other duties. Additionally, most of these therapists found that PF was unsuitable for short-term (i.e., up to 12 sessions) treatments and thus considered it not worthwhile to use:

"I think four months is very short. So, you would have ten sessions with someone, for example. And then it is hardly worth it." [Participant 9, non-user, female, age range: 31-to-40 years]

### Theme Three: Organizational Impediments

This theme concerns how therapists perceived the organization's role, both within the team culture and at a higher level. Therapists in both groups indicated strongly that the organization paid insufficient attention to PF and failed to integrate PF into the organization’s directive. Almost all the therapists emphasized the importance of proper integration, but the therapists who did not use PF expressed a stronger need for integration. All of the therapists who did use PF stressed the importance of including PF as a topic for discussion within the teams.

The *non-users* mentioned management's focus on the number of patients required to complete the questionnaires, and it highlighted potential financial repercussions in case of non-compliance. The *non-users* found this situation unpleasant and demotivating. They would have preferred that emphasis be placed on how using PF would benefit patients’ treatment. These therapists felt the need for encouragement:

"I know my responsibilities, and I would prefer for management to try to motivate and inspire me. However, the question is, if I know the purpose and I am motivated or inspired, the numbers will naturally follow." [Participant 4, user, female, age range: 31-to-40 years]

### Theme Four: Patients’ Responses and Characteristics

This topic deals with patients' responses to PF and their corresponding characteristics. Almost all the therapists experienced resistance from patients in completing the questionnaires, with *non-users* giving more reasons for why patients were not suitable for PF. They cited factors such as the lack of computer skills, lower intelligence, language difficulties, and complex situations:

"I also notice that it depends on the patient. I had an older Moroccan man, around 65, who could hardly speak Dutch and could not read Dutch either. So, in that case, I did not ask him to fill it out." [Participant 7, non-user, female, aged > 60 years]

### Theme Five: Therapists’ Familiarity with PF

This topic is related to the therapists’ familiarity with PF and the related questionnaires and how their knowledge about them had been acquired. All of the therapists reported that they had limited knowledge of the questionnaires, for example, about their reliability and validity and how results should be interpreted:

"And the visual representation, that often speaks for itself. But at the same time, you do not know what the maximum score means or what this score means, what the cut-offs are, and so on." [Participant 6, user, female, age range: 20-to-30 years]

Almost all of the therapists mentioned that using PF was an obligation imposed by the health insurers. This led to additional motivation for some therapists to learn more, but it demotivated others. Most of the therapists who used PF knew about its importance based on scientific research, whereas none of the therapists who did not use PF mentioned this. Two therapists who used PF indicated that they had learned about it during their postgraduate education.

"I think that, well, I learnt that during my training. Of course, I just finished it, and you hear at college that it is important. You hear about biases in how you do things as a therapist." [Participant 6, user, female, age range: 20-to-30 years]

When asked about their reasons for using PF, the *users* highlighted the influence of supervision and practices at their previous workplace:

"Yeah, because it was done at ... [names workplace]. I think, honestly, because it was used there, so I just took it with me. They found it essential to monitor it well, to keep an eye on it, to follow up if it wasn't done." [Participant 10, user, female, age range: 20-to-30 years]

### Theme Six: Therapists’ Need for Information

This topic concerns therapists' need to obtain information about, or the added value of, PF and the related questionnaires. An unforeseen theme that emerged was therapists’ need for information and education. In particular, the therapists who did not use PF expressed a stronger desire for guidance, information, and inspiration regarding PF, than those who did use PF. For example, the *non-users* wanted to learn more about PF and its added value, and how it could be used in treating patients.

"Yes, I think having more time and training for it is essential, especially when you start a job and encounter it for the first time. It should be well-explained. Maybe some general explanation and examples would help, something that makes it more visual, or just with practical examples from the field; I think that would be more relatable and make it stick with me. I need to be shown how it is essential.'" [Participant 8, user, female, age range: 31-to-40 years]

Both the *users* and the *non-users* wanted concise and straightforward information about the questionnaires, such as which relevant questionnaires are available, what they measure, and how they should be interpreted. They also wanted these details provided in a central location:

"Yeah, I just started googling [laughs]. I could find some information on the department's drive, but not everything. It would be nice to have a go-to folder with a brief explanation of the scores, what different score ranges mean, and which questionnaires fit with which treatments. I had to do it myself, yeah, look into protocols, and google it." [Participant 6, user, female, age range: 20-to-30 years]

### Theme Seven: The Computer System’s Lack of User-friendliness

This theme is about the extent to which the computer system had been taken into account and adapted to the needs of the therapists who work with PF. Therapists who used PF praised the positive visual presentation of the questionnaire results. Both the *users* and the *non-users* appreciated the advantage of being able to submit the questionnaires digitally. However, each of the therapists mentioned the system’s lack of user-friendliness as a disadvantage, and they described the complexity, the many questionnaires, the numerous steps involved, and the absence of system notifications.

"I find it a bit user-unfriendly because, you know, I often do not know this or that. For example, I would like the IDS to be administered very often . . .. There are just countless options that make it a bit unclear. And I also have to click three or four times, too many in my opinion, to see something. That also irritates me." [Participant 4, user, female, age range: 20-to-30 years]

### Study 2

In examining the data on how therapists used PF, four themes emerged: *supporting actions to discuss PF*, *discussing PF with patients*, *modifications in the ongoing treatment*, and *peer consultation*. Subthemes are described below in italics and are clarified with quotations.

### Theme 1: Supporting Discussions of PF

This theme encompasses the different types of actions therapists reported taking to discuss PF with their patients. *Preparation Time* prior to using PF could range from less than five minutes to 20 min. This time was used to study the completed questionnaire. Therapists also prepared the questionnaires for display on the computer screen to review them with the patient during the evaluation.

*Internal Processing* involved thinking about and hypothesizing which steps should be taken next in the treatment and whether changing the intervention or the treatment plan would be necessary. In addition, the therapists also hypothesized about the origin or cause of the patient’s complaints and how to investigate what the cause might be. Therapists tended to focus mainly on patient factors (for example, whether anything had changed in their personal lives) instead of on treatment or therapist factors (for example, whether the current intervention matched the goal of the therapy or whether it affected the therapeutic relationship).

"To what extent does it also have to do with the patient himself? . . .. What is the context? What are the patient's circumstances?" [Participant 3, male, age range: 41-to-50 years]

*Providing Information to the Patient* means providing standardized information, such as when the questionnaires would be sent and when the evaluation would occur. *Specific Actions* were also taken to be able to discuss PF, e.g., to show the results from the questionnaire to the patient. To be able to do so, the therapies moved their desk and turned the monitor so that the graphics were visible to the patient.

"If I look purely at the OQ, for example, I always scroll straight to the graph to see the progression. I think there are four little graphs or so, so one total and three other scores." [Participant 6, female, age range: 21-to-30 years]

Therapists’ use of *Documentation,* e.g., forms were used during the evaluations and the intakes, and they were also used to guide the conversations about PF. The therapist incorporated conclusions drawn from the conversation into the documentation, e.g., in reports, a letter to the referrer, and the treatment plan.

*Time Spent Discussing PF* showed considerable variation: It took five minutes when nothing unusual emerged from the questionnaires, but longer than 20 min when the patient’s symptoms had not ameliorated or had intensified. The better the process of discussing PF was embedded in the therapist's team, the more time was devoted to discussing PF.

### Theme 2: Discussing PF with the Patient

This theme includes the variety, depth, and range of ways in which PF was discussed. *Discussing Substantive Information with the Patient* was done at a global level using both the overall score and the subscale scores and individual items. The questionnaire results were compared with the normative scores and used to objectify any complaints. Participants emphasized that they placed little or no value on the outcome of the questionnaire per se. Instead, they used the results from the questionnaire to explore, together with the patient, what the scores meant and how they should be interpreted.

"[I] still tend to shift the focus slightly from those questionnaires because I do not see the added value at that point anyway. Because I think that I find the added value of directly discussing what I can see is happening and that I then find a questionnaire more of a distraction from the thing that matters, rather than adding anything. And in an evaluation, so looking at the big picture, I think it has a slightly greater value [to discuss the questionnaire]. Because then you are looking at the process as a whole." [Participant 7, female, age range: 21-to-30 years]

"I do really believe it is important to discuss progress. I do ask a lot more often these days, ‘How was the session for you?’ ‘Did we work on your problem in a good way?’" [Participant 8, male, age range: 31-to-40 years]

During the conversation about PF, various topics and ways to discuss them emerged that contributed to *Forming/Testing a Hypothesis*, e.g., when there was a discrepancy between what the patient said and what the questionnaire results showed. The therapist invited patients to share their ideas and hypotheses and then checked whether the questionnaire results were meaningful for the patient. Finally, the therapists shared their own hypotheses.

"I see that symptoms regarding your mood have not completely . . . cleared up. Do you experience that as well? Then I indicate [that it] would be my idea to focus more on that and ask them what they think." [Participant 3, male, age range: 41-to-50 years]

"And that is what the conversation was about as well: That is quite an improvement over the last time we spoke . . .. What do you think helped the most?" [Participant 3, male, age range: 41-to-50 years]

*Evaluations* were mainly used to pinpoint the focus of the treatment. The therapist monitored patients’ progress, discussed with patients what contributed to their progress, and linked treatment interventions to skills acquired.

"Does that still affect what we are doing? Do we have the right focus? Are we doing the right therapy?" [Participant 4, female, age range: 21-to-30 years]

"In doing so, I started the conversation by . . . saying, 'Gosh, we are here to evaluate, so I want to ask you to look back at the past period. What has improved, what has changed, what did you overcome? I would also like to look ahead together: What do you need? How do you want to go about reaching your goals?" [Participant 10, female, age range: 41-to-50 years]

Finally, PF was used as an *Intervention*: the therapist added another purpose to the conversation, such as motivating patients and validating them. PF was also used when treatment was stagnating.

"What I do is to put the patient into a position to take more control, to take the lead." [Participant 3, male, age range: 41-to-50 years]

"If I know [that] the patient knows what his condition is, what his fever is, I start with that. Because that is, in fact, the first thing the patients assume, that such a questionnaire works the same as a thermometer. Then that will develop into something we do together: what we talk about together, what we evaluate together, and [what] the progress should be. And the third step [is that] patients [use] the ROM to evaluate themselves and take ownership of the evaluation of the process." [Participant 5 male, age range: 60+ years]

### Theme 3: Modification of Ongoing Treatment

This theme contains therapists' preferences for modifying the treatment from what they had been doing. Changing the ongoing treatment was done in different ways, namely by changing the treatment plan, intensifying the treatment, or terminating the treatment. Intensification included upscaling the treatment from basic to specialized mental health care. Finally, interventions were fine-tuned or added to without substantive changes to the overall treatment plan.

### Theme 4: Peer Consultation

Another way in which PF could be improved was through the use of various types of peer consultation. The most frequently mentioned type of peer consultation was for the supervisor to discuss ways in which to avoid therapists’ inability to ameliorate patients’ symptoms. In addition, peer review with colleagues was frequently mentioned as an opportunity to utilize peer consultation to discuss PF.

Discussing PF in the multidisciplinary peer consultation was often mentioned by some participants, which implied a connection with the level of inclusion in a team. Compared to multidisciplinary peer consultation, supervisors discussed PF with their supervisees to a lesser extent. If this happened at all, it was because of treatment stagnation or deterioration.

"When deterioration occurred, I discussed it with my supervisor, and then [we] talked about whether treatment intensification early on [was needed]. If more was needed, more therapists were also needed. Or . . . whether maybe we should use different interventions." [Participant 9, female, age range: 21-30 years]

**Fig. 1:**
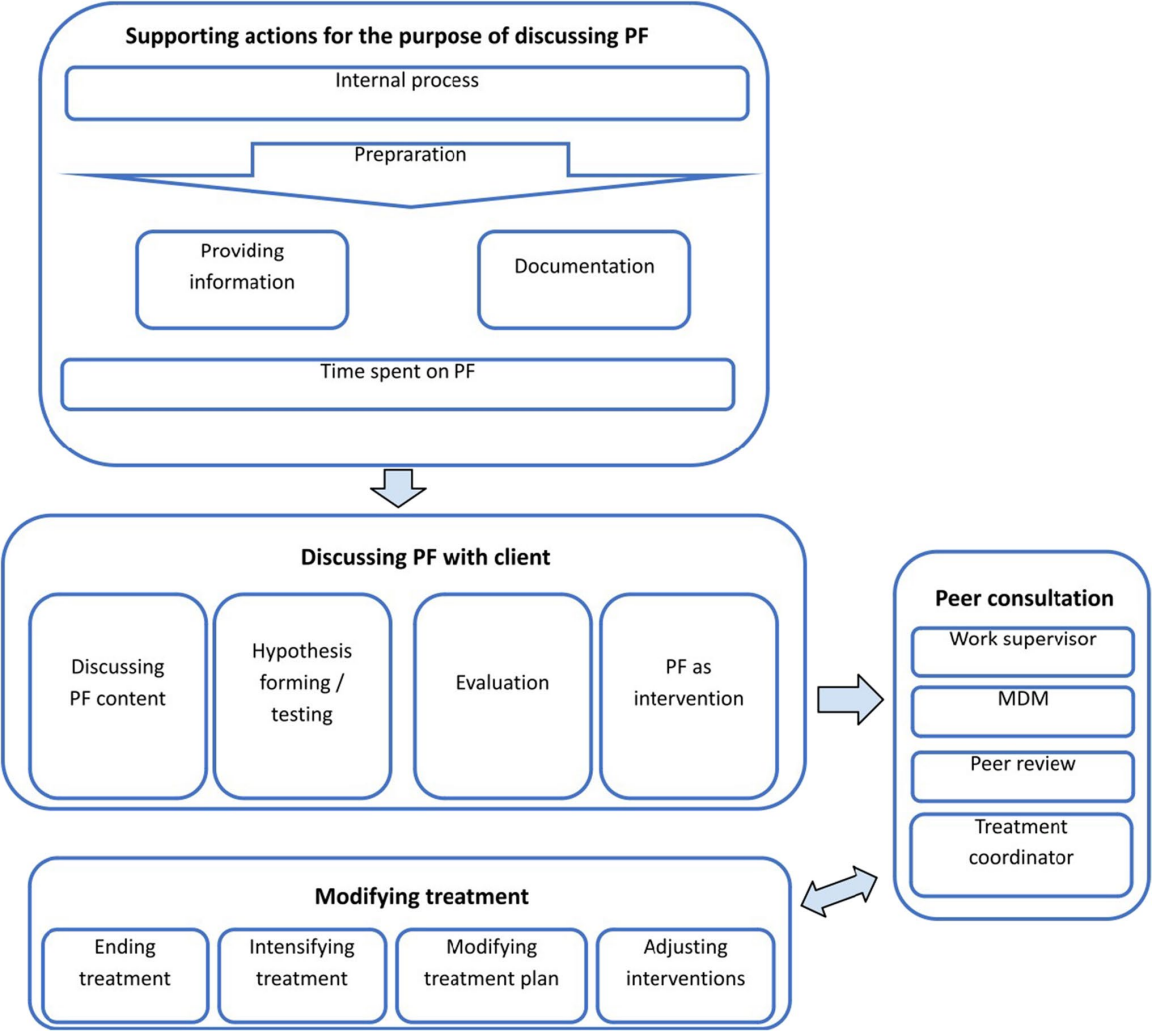
Visual representation of how therapists use progress feedback. *Note*. MDM = Multidisciplinary (peer consultation) meeting

A summary of the themes and subthemes discussed above is presented in Fig. 1. Besides providing a schematic representation of the relationship between the themes, this figure also shows how the themes and subthemes are related as well as the order in which actions occurred. The various supporting actions for the purpose of discussing PF precedes the actual discussion of PF with the client, after which peer consultation takes place. Peer consultation in turn can lead to the modification of the treatment plan, whereupon peer consultation can also take place again.

## Discussion

In these two qualitative studies, we investigated the attitudes and motivations of therapists who used or did not use PF. Our aim was to gain more insight into psychologists' utilization of PF.

The most striking result from the first study was that all therapists, irrespective of their use of PF, had a positive attitude about PF. Both the therapists who used PF and those who did not perceived PF as a valuable tool to use in treatment. They perceived PF as contributing to information gathering and treatment evaluation, which is in line with earlier research (Norman et al., 2014; Unsworth et al., 2012). Among the questionnaires, therapists favored those targeting PTSD symptoms (the PCL-5) and general symptomatology, relationships, and social roles (the Outcome Questionnaire-45). However, a shared concern was therapists’ limited understanding of PF questionnaires. They indicated a need to acquire comprehensive information on reliability, validity, and applicability. They also expressed a need for centralized, accessible resources.

The therapists who used PF and those who did not use it differed in their knowledge about PF’s potential. Non-users lacked insight into PF's benefits for treatment, whereas users clearly recognized its value. Users mentioned treatment monitoring and treatment adjustments as the main incentives for using PF. Such practices have proven to be effective in enhancing treatment outcomes (De Jong et al., 2021). Therapists who used PF often showed scientific insight, acquired during their postgraduate education, into PF's therapeutic contributions. This was in line with previous research results; For example, Williams et al. (2022) found that therapists who were exposed to PF during their postgraduate training were more likely to have a positive attitude about using PF. Non-users, on the other hand, expressed reservations about PF's suitability for use in short-term treatments, possibly due to their limited knowledge of its efficacy, as PF has been shown to be particularly beneficial only in the initial months of therapy (Bovendeerd et al., 2022). Non-users also cited various patient-related reasons for PF's inappropriateness. The lack of information coincided with non-users’ expressed desire for more information about and enthusiasm for PF's applications. What is important for implementing PF and embedding it into an organization is that providing information about PF should not be a one-time event; instead, there should be an ongoing process of informing and consulting (Marriott et al., 2023). As Wray et al. (2023) showed, intensive facilitation of implementation of progress feedback in teams can help improve its uptake.

Among the therapists who used PF, its adoption into their current practice had been facilitated by its prominence in their work supervision and early training, consistent with other settings emphasizing evidence-based treatment (Casline et al., 2023; Williams et al., 2022). However, the organizational integration of PF was found to be lacking, particularly in the view of the non-users. Implementation of PF seemed financially motivated and focused on completion rates and other financial implications rather than on the therapeutic benefits. This proved to be demotivating for the non-users. Accordingly, the non-users sought encouragement based on content-driven insights into PF's contributions to treatment outcomes.

In sum, this study included both therapists who used PF and those who did not. It confirmed that although the users had a positive attitude about PF, the non-users lacked essential information and sought guidance. Both groups highlighted the need for easily accessible information.

The second study showed that psychologists employed various strategies to facilitate discussions of PF with their patients. Consistent with prior research (Hovland & Moltu, 2019; Hovland et al., 2020), participants valued visual representation of the questionnaire results through graphs and color-coded severity indicators. When scores on the questionnaires did not change or indicated that patients’ symptoms were worsening, the therapists explored possible explanations with their patients, actively inquiring about patients’ perspectives and sharing their own views. Therapists collaboratively evaluated patients’ treatment progress and planned the next steps in the treatment, sometimes using PF as an empowering intervention, which enabled patients to assume more control.

Despite the therapists’ discussions of PF with their patients, they indicated that they rarely made adjustments in the treatment plans. Also, PF was rarely discussed in peer consultation meetings despite the possibility that this would have been beneficial (Sun et al., 2021). One reason why PF was not used to change treatment strategies or discussed in peer consultation meetings is that the setting in which the therapists worked encouraged strict adherence to treatment guidelines and protocols. Although management discussed using PF, having the flexibility to adapt treatment plans based on PF was not emphasized. This possibly affected the delivery of personalized care and the adaptation of treatment plans based on PF. Additionally, focusing on preventing *therapist drift* (Waller & Turner, 2016) might have impeded adjusting treatment plans based on PF.

Despite rigorous sample selection, all the participants were affiliated with the same institution, and they delivered similar interventions with comparable patient groups who were accustomed to PF. Therapists' attitudes about and experiences with PF in other mental health settings might differ due to varying treatment approaches used and different patient needs. These factors could affect transferability to other settings or populations. Furthermore, despite keeping a sample matrix and conducting targeted sampling, most of the participants were female, so that the groups were less heterogeneous than ideal. Previous research by De Jong et al. (2012) has shown that women are more likely to use PF than men. Moreover, the findings depend on participants' self-reports, memories, and perceptions of their application of PF. There might also have been discrepancies between self-reports and actual behavior. For instance, therapists might discuss PF more or discuss it less than they recalled.

Future research could extend this study's insights by using video observations, which would allow objective coding of therapists' behaviors. In addition, following the recorded session, the recording could be reviewed with the respective practitioner to understand their thought process during the conversation. Additionally, incorporating patient interviews into the protocol might verify therapists' claims and allow examination of patients’ perceptions of adjustments in the treatment based on PF. Further exploration of the impact of adherence to treatment integrity on interventions or adjustments in treatment plans is also warranted.

In conclusion, the findings of the two studies provide insights into how the integration of PF into therapeutic practices might be enhanced. Recommendations for how to achieve this goal include early and ongoing education about PF, greater accessibility of information about relevant questionnaires, integration within organizations, more discussion of PF in peer consultations, adapting treatment plans when needed, and an increased focus of management on the therapeutic value of PF.

## Supplementary Information

Below is the link to the electronic supplementary material.

Supplementary file1 (DOCX 14 KB)
