# Correction to: Motives of Therapists for Using Routine Outcome Monitoring (ROM) and How it is Used by Them in Clinical Practice: Two Qualitative Studies

**DOI:** 10.1007/s10488-024-01383-1

**Published:** 2024-05-16

**Authors:** Shaghayegh Azizian Kia, Lisette Wittkampf, Jacobine van Lankeren, Pauline Janse

**Affiliations:** 1 Pro Persona Research, Wolfheze, The Netherlands; 2 Behavioural Science Institute, Radboud University, Nijmegen, The Netherlands

**Correction to: Administration and Policy in Mental Health and Mental Health Services Research**

10.1007/s10488-024-01374-2

In this article, the author Dr. Azizian Kia given name “Shaghayegh” was incorrectly written as “Sharaya”.

That is, Sharaya Azizian Kia should be Shaghayegh Azizian Kia.

The original article has been corrected.

