# Supplementary material for: Motives of Therapists for Using Routine Outcome Monitoring (ROM) and How it is Used by Them in Clinical Practice: Two Qualitative Studies
Source: Adm Policy Ment Health. 2024 Apr 8;52(1):159–70. doi: 10.1007/s10488-024-01374-2 (PMC11703879; doi:10.1007/s10488-024-01374-2)
Supplement: Supplementary file 1 — Supplementary file1 (DOCX 14 KB) [file 10488_2024_1374_MOESM1_ESM.docx]

| Table 1. Key interview guide questions Study 1 | |
| --- | --- |
| **Interview guide 'users'** | |
| *How do you experience using progress feedback?* |  |
| *What was the reason for starting to use PF?* |  |
| **Interview guide 'non-users'** | |
| *Have you used or encountered progress feedback earlier in your career?* |  |
| *How did you experience using progress feedback?* |  |
| *What are your considerations for not (or no longer) using progress feedback?* |  |
| *When would you start using it (again)?* |  |

| Table 2. Key interview guide questions Study 2 | |
| --- | --- |
| *What do you do with the information you get from PF?* | |
| *How do you prepare for the session regarding PF?* |  |
| *Is there a specific way you present data from questionnaires when discussing PF?* |  |
| *In case of stagnation, how do you use information from PF to alter the treatment plan or interventions?* | |
| *Do you discuss information obtained regarding PF in peer review? If so, how?* |  |
| *What do you do with the information you get from PF?* |  |
